# Derivation of Pluripotent Cells from Mouse SSCs Seems to Be Age Dependent

**DOI:** 10.1155/2016/8216312

**Published:** 2015-11-09

**Authors:** Hossein Azizi, Sabine Conrad, Ursula Hinz, Behrouz Asgari, Daniel Nanus, Heike Peterziel, Akbar Hajizadeh Moghaddam, Hossein Baharvand, Thomas Skutella

**Affiliations:** ^1^Institute for Anatomy and Cell Biology, Medical Faculty, University of Heidelberg, Im Neuenheimer Feld 307, 69120 Heidelberg, Germany; ^2^Department of Stem Cells and Developmental Biology at the Cell Science Research Center, Royan Institute for Stem Cell Biology and Technology, P.O. Box 19395, Tehran 4644, Iran; ^3^Faculty of Veterinary Medicine, Amol University of Special Modern Technologies, P.O. Box 49767, Amol 49767, Iran; ^4^P.O. Box 12 43, 72072 Tübingen, Germany; ^5^Division of Signal Transduction and Growth Control, DKFZ/ZMBH Alliance, Heidelberg, Germany; ^6^Department of Biology, Faculty of Basic Sciences, University of Mazandaran, Babolsar, Iran; ^7^Department of Developmental Biology, University of Science and Culture, ACECR, Tehran, Iran

## Abstract

Here, we aimed to answer important and fundamental questions in germ cell biology with special focus on the age of the male donor cells and the possibility to generate embryonic stem cell- (ESC-) like cells. While it is believed that spermatogonial stem cells (SSCs) and truly pluripotent ESC-like cells can be isolated from adult mice, it remained unknown if the spontaneous conversion of SSCs to ESC-like cells fails at some age. Similarly, there have been differences in the literature about the duration of cultures during which ESC-like cells may appear. We demonstrate the possibility to derive ESC-like cells from SSC cultures until they reach adolescence or up to 7 weeks of age, but we point out the impossibility to derive these cells from older, mature adult mice. The inability of real adult SSCs to shift to a pluripotent state coincides with a decline in expression of the core pluripotency genes Oct4, Nanog, and Sox2 in SSCs with age. At the same time genes of the spermatogonial differentiation pathway increase. The generated ESC-like cells were similar to ESCs and express pluripotency markers.* In vitro* they differentiate into all three germ lineages; they form complex teratomas after transplantation in SCID mice and produce chimeric mice.

## 1. Introduction

Pluripotent stem cells (PSCs) are undifferentiated cells which have the potential for proliferation, self-renewal, and differentiation into ectodermal, mesodermal, and endodermal cells of all three embryonic germ layers* in vitro* and* in vivo* [1]. So far, several different approaches were used for the generation of PSCs, including ESCs obtained after fertilization from the inner cell mass of an embryo at the blastocyst stage [1, 2]. They were also procured by enforced expression of pluripotency genes in somatic cells, giving rise to the so-called induced pluripotent stem cells (iPSCs) [3, 4]; one of the promising methods for a more natural and ethical unproblematic establishment of PSCs is SSCs, especially for therapeutic approaches in human medicine [5–11]. SSCs are present in a small number in the testis, but they can be isolated and expanded* in vitro* [5]. Although they are unipotent stem cells under the environmental control of their stem cell niche, under specific culture conditions outside the niche and without any exogenous pluripotency genes, they are able to convert to ESC-like cells at different times after the initiation of culture or isolation of SSCs [5, 9, 10].

The generation of PSCs of mouse testis cells dates back to 2004 by Kanatsu-Shinohara et al. [5], when they generated ESC-like cells in SSC culture from two-day-old pups and obtained these cells 4–7 weeks after the initiation of culture. Guan et al. [9] obtained ESC-like cells from populations of STRA8-GFP positive cells of 4–7-week-old adult mice. Ko et al. repeated the induction of pluripotency in 5-week- to 7-month-old Oct-4 GFP positive adult SSCs and described the dependence of the induction on the initial number of plated SSCs and the length of culture time of Oct-4-positive cells without splitting [7]. On the other hand, this group worked in the later published protocol of conversion of SSCs into pluripotent stem cells only with SSCs of mice from postnatal day 35 (5 weeks old) [8].

Also Seandel et al. generated adult spermatogonial-derived stem cells from GPR125-positive cells in 3-week- to 8-month-old mice, but these cells were only multipotent [10].

In our experiments, we identified the spontaneous conversion of SSCs in ESC-like cells from neonate and nearly adult testis up to 7-week-old mice. On the contrary, it was impossible to generate ESC-like cells from mice older than 7 weeks. According to the NIH criteria (http://www.researchgate.net/post/At_what_age_are_laboratory_mice_considered_adult2), mice are considered adult after 8 weeks of age. The sexual activity of mice starts between 5 and 6 weeks of age [12]. According to Finlay and Darlington [13], mice should be considered mature adult between 3 and 6 months of age.

The potential generation of pluripotent cells from SSCs can apparently only be realized up to the age of 7 weeks. Therefore, it is a debatable point whether generation of pluripotent SSCs depends on their development status in correlation with the completion of puberty. The possibility of generating ESC-like cells from this cell type seems to stall before donor mice are fully matured adults.

## 2. Material and Methods

### 2.1. Isolation of SSCs and Establishment and Culture of ESC-Like Cells

All animal experiments were confirmed to the local and international guidelines for the use of experimental animals and were approved by the Royan Institutional Review Board and Institutional Ethical Committee (Tehran, Iran) and by the regional authorities in Germany (Regierungspräsidium Karlsruhe). Testis cells were isolated from C57BL/6, 129/Sv mouse strains of 6-day- to 6-month-old transgenic Oct4-GFP-reporter mice. After removing the tunica albuginea, the seminiferous tubules were separated and placed in a digestion solution which contained collagenase IV (0.5 mg/mL, Sigma), DNAse I (0.5 mg/mL, Sigma), and Dispase I (0.5 mg/mL, Roche) in HBSS buffer with Mg^++^ and Ca^++^ (PAA) at 37°C for 8 minutes. Digestion enzymes were stopped with 10% ESC-qualified FBS (Invitrogen) and additionally the cell suspension was triturated by pipetting to obtain a single cell suspension. After centrifugation, the cell pellet was washed with DMEM/F12 (PAA), filtered through a 70 *μ*m cell strainer, and centrifuged again for 10 minutes at 1200 rpm. The supernatant was completely removed and the cells were resuspended in mouse SSC medium (StemPro-34 medium, N2-supplement, D+ glucose, bovine serum albumin, L-glutamine, *β*-mercaptoethanol, penicillin/streptomycin, MEM vitamins, NEAA, estradiol, progesterone, EGF, FGF, GDNF, LIF, ES-FBS, ascorbic acid, pyruvic acid, and DL-lactic acid) and plated onto 0.1% gelatin-coated culture dishes (5 × 10^5^ cells per 9.6 cm^2^ for neonate and 5 × 10^5^ cells per 9.6 cm^2^ for adult mice). About 3–7 days later, cultures of cells from neonate mice and 7–14 days later from adult mice, GFP-positive SSC colonies were manually selected from the primary culture and plated on a mouse embryonal feeder (MEF) layer in at least four 24-well plates (approximately 500 cells per well) per group. Cells were passaged 1 : 1–1 : 4 every 3-4 weeks. At the beginning, SSCs expressed Oct4-GFP especially from neonate mice and much weaker in mice older than 7 weeks. But this signal was downregulated after 2-3 weeks after the initiation of culture. During SSC cultivation we screened daily for colonies which were similar to mouse ESCs, ESC-like cells that re-expressed a high level of Oct4-GFP.

These generated ESC-like cells were manually selected and subcultured on a MEF feeder layer in mouse ESC (mESC) medium with KO-DMEM, (Invitrogen) 15% ESC-qualified FBS (Invitrogen), 1% NEAA (PAA), 1% L-glutamine (PAA), 1% Pen-Strep (PAA), 0.1%  *β*-mercaptoethanol (Invitrogen), and LIF (ESGRO, Millipore) at a final concentration of 1000 U/mL. ESC-like colonies were grown in mESCs media and were passaged every 3-4 days.

In the supplementary method section (in Supplementary Material available online at http://dx.doi.org/10.1155/2016/8216312), we describe in more detail the material and methods of RNA extraction and RT-PCR analysis, gene expression analyses (Fluidigm Biomark), immunofluorescence staining (IMH), electrophysiology, FACS analysis, alkaline phosphatase assay, embryoid body (EB) formation, neuronal differentiation, cardiomyocyte differentiation production of teratoma and chimeric mice, and the statistical analysis.

## 3. Results

### 3.1. Isolation and Expansion of SSCs

**Figure 1:**
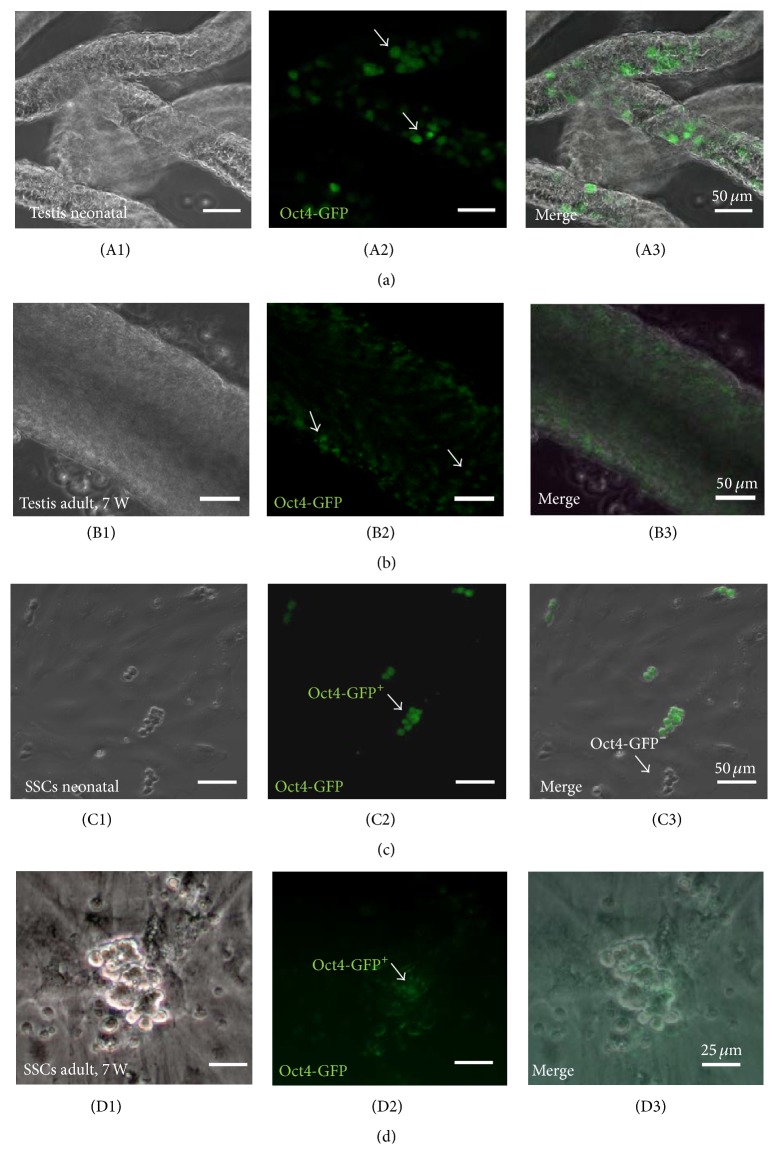
Number and intensity of GFP signals in the neonate and adult mouse testicular tubules (a, b) and SSC cultures (c, d) from transgenic Oct4-GFP reporter mice. (A1–A3) In the freshly dissected testicular tubules, the number of Oct4-GFP positive cells and the intensity of the Oct-GFP signal were higher and stronger in neonate mice than in adult mice >7 weeks (B1–B3). (C1–C3) Oct4-GFP positive SSCs were clearly present during initial cultures from neonate mice, while in adult mice >7 weeks SCCs Oct4-GFP signals were much weaker from the beginning (D1–D3). SSC colonies were grown on MEF feeder layers. (A1–D1) bright field; (A2–D2) green fluorescence for Oct4-GFP; (A3–D3) merged images. Scale bars: (a)–(c) 50 *μ*m, (d) 25 *μ*m.

After mild digestion with collagenase, the seminiferous testicular tubules from neonatal, 7-week-old, and 12-week-old mice were separated and could be microscopically investigated under UV-light. The Oct4-GFP signal was clearly observable in the freshly isolated seminiferous tubules of neonate mouse testis (Figure 1(a)), while in adult mice the number and intensity of Oct4-GFP signals were much lower and very low in 12-week-old mice (Figure 1(b)). After digesting and plating, the expression of the Oct4-GFP signal was detectable in both neonate and adult SSCs, although in adult SSCs to a much lower extend and with lower intensity (data not shown). All the isolated Oct4-GFP SSCs were positive for DDX4 (Vasa) and negative for Vimentin immunocytochemistry (data not shown). The morphology of SSCs was similar, irrespective of the age of the mice and the days of the culture. Representative examples of spermatogonial cultures are shown in Figure 1.

Up to 14–21 days after initiation of the primary testis cultures with SSC medium, SSCs with a positive Oct4-GFP signal were observed during the culture of neonate but were observed very rarely during the culture of adult mice (Figures 1(c) and 1(d)). After three weeks, the Oct4-GFP signal was completely downregulated and in the near of not observable during long-term culture (data not shown). The SSCs were passaged for more than 22 times and could be cultivated up to one year and longer.

### 3.2. Gene Expression Profiling of SSCs from Neonatal and Adult Mice

We quantified and analyzed the expression of important germ cell-enriched genes (*LHX1, Stella, VASA, DAZL, CD9, EPCAM, GPR125, GDF3, THY1, STRA8, GFRa1, 1ITGB1, TAF4b, KIT, ETV5,* and* BCL6B*) and pluripotency associated genes (*Oct4, Nanog, Sox2, TDGF1, KLF4, MYC, LIN28, SALL4, DPPA3,* and* DPPA5*) in neonatal and adult SSCs, which were obtained from 7- and 12-week-old mice by real-time PCR with Fluidigm nanofluid technology.

**Figure 2:**
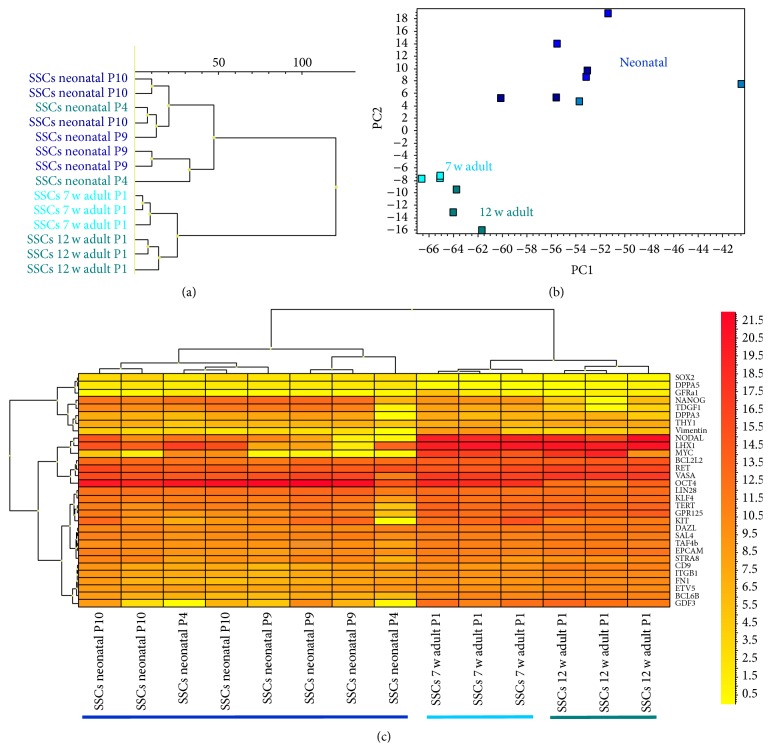
Different gene expression profiles of neonatal and adult SSCs with germ cell-enriched and pluripotency associated genes. Adult SSCs were obtained from 7- and 12-week-old mice. (a) Dendrogram and (b) PCA demonstrate that neonate and adult SSCs are distinct and localize in separated trees in the dendrogram or areas in the PCA. (c) Heat map shows array of pluripotency and germ cell associated genes with a cluster of different populations of neonatal SSCs (coloured dark blue), while adult SSCs cluster from 7- and 12-week-old mice in a separate tree (coloured light blue and green).

Hierarchical clustering (dendrogram) and principal component analysis (PCA), as in Figure 2, made evident that neonatal and adult SSCs are different and localized in separated trees in the dendrogram or areas in the PCA.

The heat map analysis with an array of pluripotency and germ cell associated genes revealed that a cluster of various populations of neonatal SSCs was significantly different from the other groups, while adult SSCs from 7- and 12-week-old mice clustered in a separate tree.

**Figure 3:**
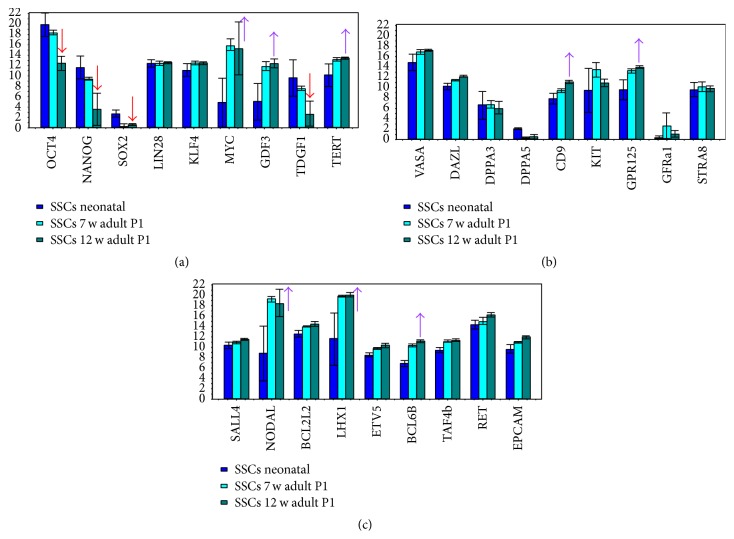
Bar plot showing expression of pluripotency and germ cell associated genes between neonatal SSCs (coloured dark blue), 7-week-old adult SSCs (coloured light blue), and 12-week-old adult SSCs (coloured green blue). Red arrows mark significantly downregulated genes and purple arrows mark upregulated genes in adult SSCs (more than 2-fold and *P* < 0.05). Note that the core pluripotency genes Oct4, Nanog, and Sox2 are downregulated in adult SSCs from 12-week-old mice.

The neonatal SSCs expressed a significantly higher level of the pluripotency* genes Oct4, NANOG, TDGF1*, and* Sox2* in comparison to adult SSCs (fold change > 2 and *t*-test *P* < 0.05) (Figures 2 and 3; Supplementary Table 1).

In contrast, several germ cell associated genes in the adult SSCs were expressed in descending order* MYC, NODAL, LHX1, GDF3, GPR125, BCL6B, TERT, CD9, ITGB1,VASA, TAF4b, EPCAM, BCL2L2, ETV5, DAZL, KLF4, RET*, and* THY1* and at a significantly higher level than in neonatal SSCs (fold change > 2 and *t*-test *P* < 0.05).

Not significantly regulated between neonate and adult SSCs were* GFRa1, KIT, STRA8, LIN28*, and* DPPA3* (fold change > 2 and *t*-test *P* < 0.05).

In a comparison between neonatal SSCs and SSCs obtained from 12-week-old mice, these differences became even more apparent (see Supplementary Tables). Moreover, comparing SSCs from 7-week-old and 12-week-old mice, the pluripotency genes are significantly higher expressed in SSCs obtained from 7-week-old mice.

**Figure 4:**
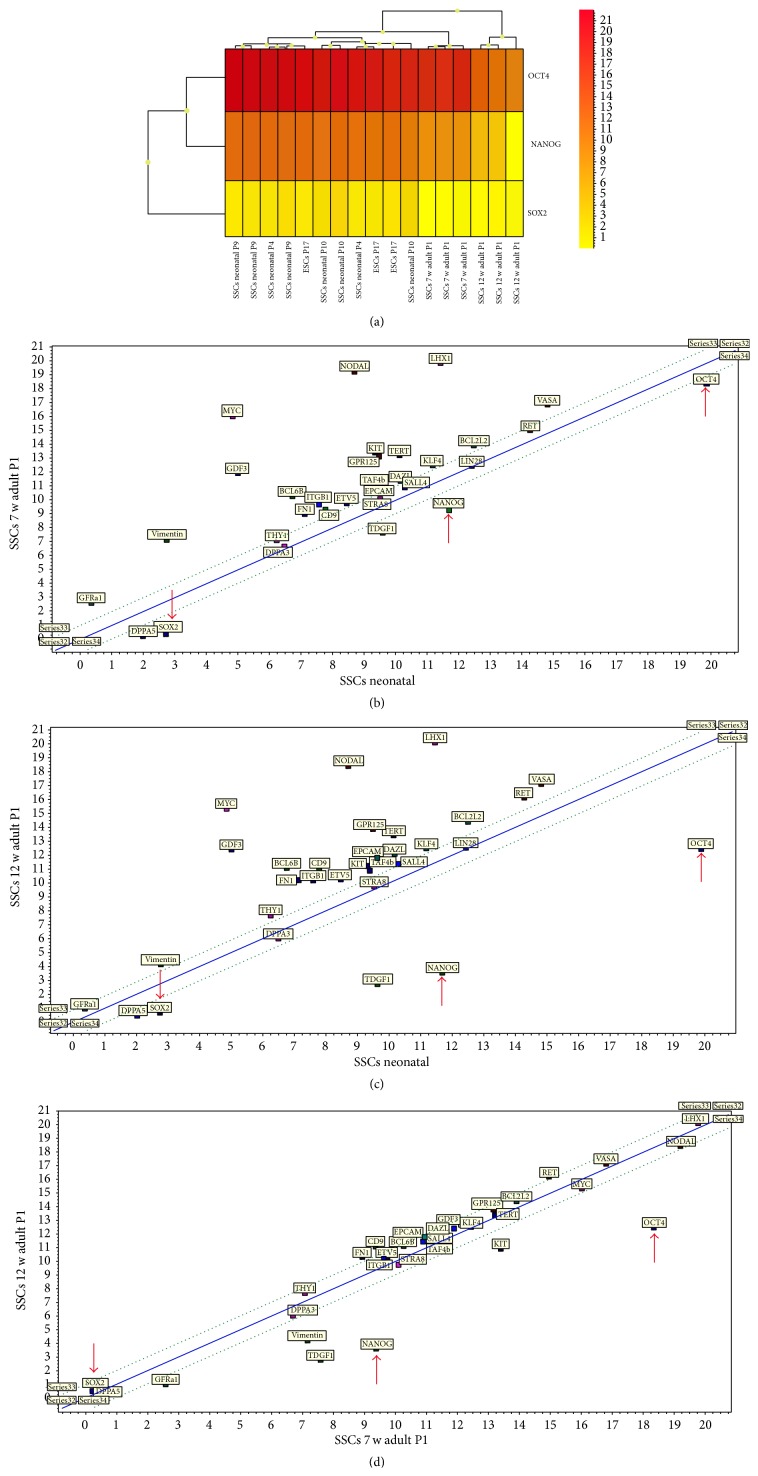
(a) Heat map and (b–d) correlation analyses reveal expression levels of core pluripotency genes Oct4, Nanog, and Sox2 decrease in SSCs with age of the animal. A decline in Oct4, Nanog, and Sox2 expression is clearly observable after 7 weeks of age and becomes even more evident after 12 weeks of age. Arrows in (b–d) mark the localization of Oct4, Nanog, and Sox2 in the correlations.

As apparent in the bar plot (Figure 3), the core pluripotency genes Oct4, Nanog, and Sox2 were significantly downregulated in adult SSCs from 12-week-old mice, while the expression of germ cell genes was found more stable in the more developed and differentiated epithelium of spermatogenesis. In contrast to neonatal SSCs and those from 7-week-old mice, Oct4 was also insignificantly differentially expressed in 12-week-old mice in comparison to fibroblasts. This possibly indicates that an important prerequisite for a natural shift to pluripotency in male germ cells is lost during adolescence. The decline in the expression of pluripotency genes at the edge of adultness is demonstrated in aheat map and correlation analyses in Figure 4 as well. The expression levels of core pluripotency genes* Oct4, Nanog*, and* Sox2* decrease in SSCs with the age of the animal.

### 3.3. The Occurrence of Pluripotent ESC-Like Cells from Oct4 GFP Positive Cells Is Restricted to Neonatal up to 7-Week-Old Mice

**Figure 5:**
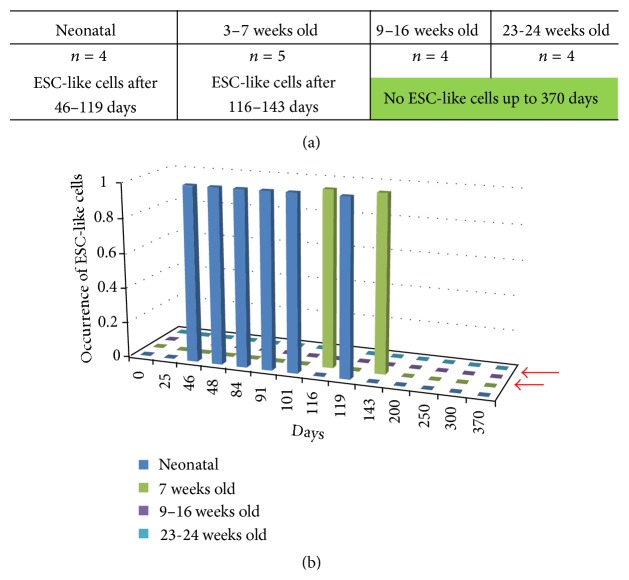
(a) Table and (b) graph demonstrate that ESC-like cells can only be obtained with SSCs obtained from mice until the age of 7 weeks. SSCs obtained from older adult animals are unable to show a shift to pluripotency (red arrows).

**Figure 6:**
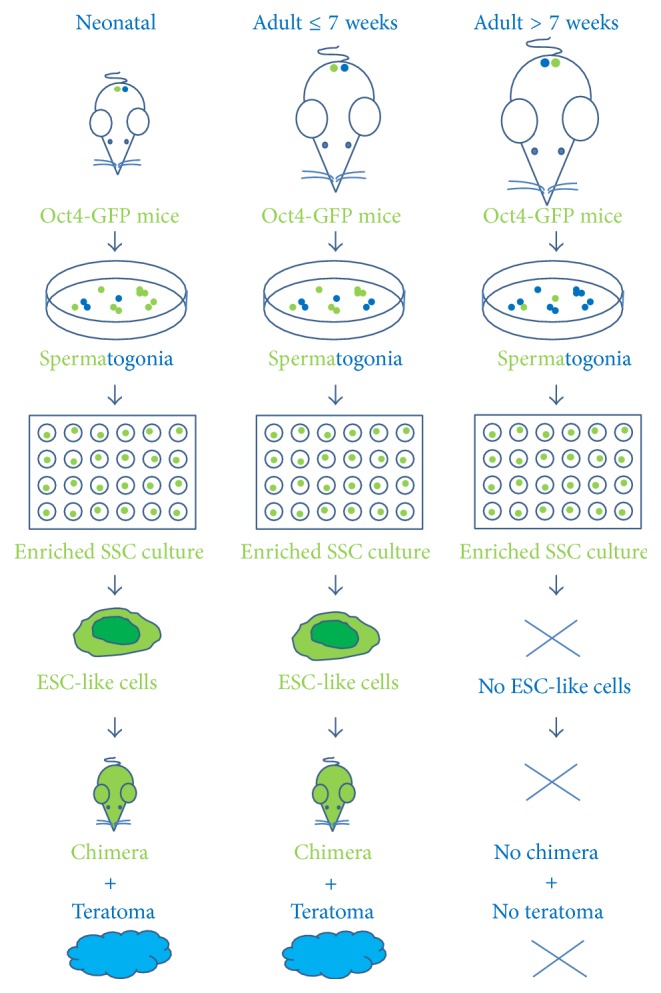
Schematic illustration pinpoints that occurrence of pluripotent ESC like cells from Oct4 GFP positive cells with the production of chimera and formation of teratoma is restricted to neonatal up to 7-week-old mice.

As documented in Figures 5 and 6, ESC-like cells could only be generated from SSCs obtained from mice not older than 7 weeks, during a cultivation time of these SSCs between 46 days and 143 days. As shown in Figures 5(a) and 5(b), ESC-like colonies were observed in days 46, 48, 84, 91, 101, and 119 in neonatal SSCs and after 116 and 143 days in 7-week-old mice, counting from initiation of the culture (Figures 5 and 6). No ESC-like cells or colonies could be observed in the groups of SSCs obtained from 9–16-week-old and 23-24-week-old mice (Figures 5 and 6).

We observed no development of ESC-like colonies from the SSC cultures at all before 46 days and after 143 days.

### 3.4. The ESC-Like Cells Are Fully Pluripotent, Form Teratoma, and Produce Chimera

**Figure 7:**
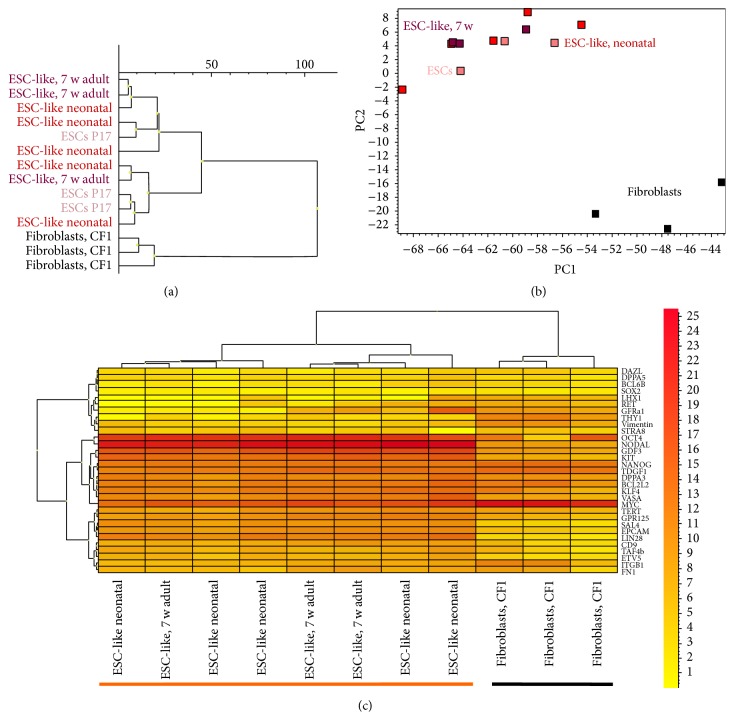
Similar gene expression profiles of ESC-like cells and mESCs with germ cell-enriched and pluripotency associated genes. (a) Dendrogram and (b) PCA clearly demonstrate that ESC-like cells and mESCs are similar to each other but distinct to fibroblasts which localize in separated trees or areas. (c) Heat map shows array of pluripotency and germ cell associated genes with a cluster of ESC-like cells and mESCs (both underlined with orange bar), while fibroblasts cluster in a separate tree (underlined with black bar).

**Figure 8:**
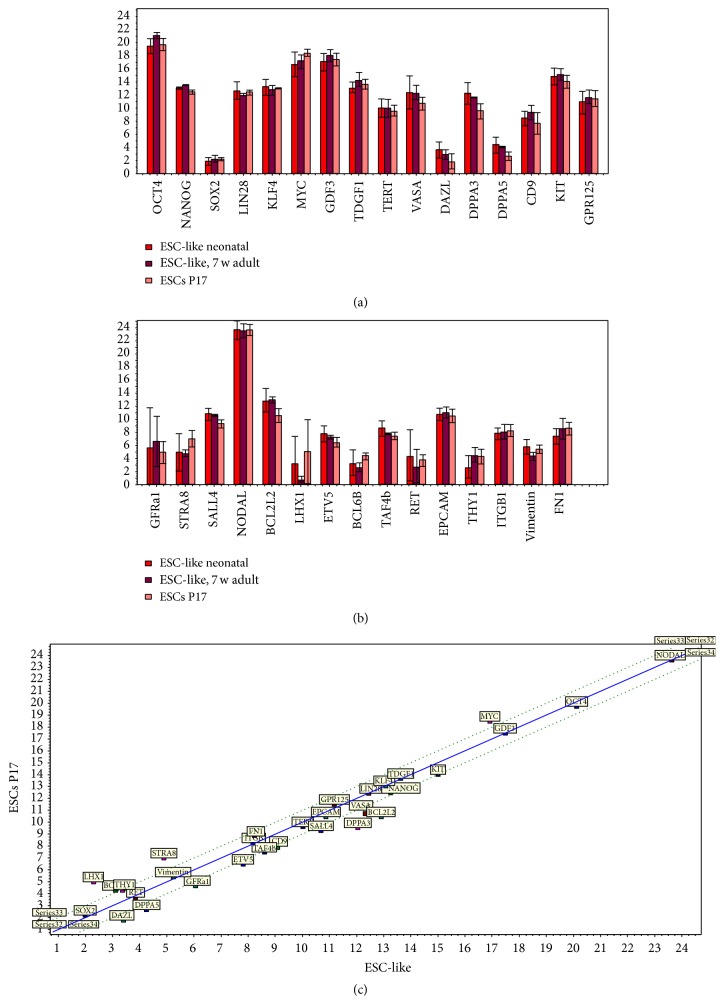
Bar plot showing expression of pluripotency and germ cell associated genes between ESC-like cells (coloured pink or dark red) and mESCs (coloured light red). Note that the core pluripotency genes Oct4, Nanog, and Sox2 are not differentially regulated between ESC-like cells and mESCs.

ESC-like colonies had a packed spindle- to round-shaped morphology with smooth borders (Supplementary Figure 2A1). Moreover, they displayed a high intensity of the Oct4-GFP signal (Supplementary Figures 2A2, 2A3). The ESC-like cell lines were passaged 1 : 5–1 : 8 for more than 15 times following trypsin digestion, with an estimated doubling time of 48–72 h. They still expressed Oct4-GFP after long-term cultivation. The cells preserved their undifferentiated state in multiple passages. The established ESC-like cell lines were successfully expanded, cryopreserved, and thawed with no loss in proliferation or differentiation capacities. Figures 7 and 8 make evident the close similarity of the gene expression profiles of ESC-like cells and ESC with germ cell-enriched and pluripotency associated genes but show clear distinction to fibroblasts. Reading the dendrogram, PCA, and heat map, we observe the ESCs derivation from neonatal and 7-week-old mice and the ESCs intermingling in den trees und areas. Moreover, it becomes evident from the bar plots that all of the pluripotency genes were strongly expressed in ESC-like cells and ESCs (Figures 7 and 8; Supplementary Tables 1 and 2).* DPPA3, BCL2L2, SALL4*, and* Nanog* were upregulated in ESC-like cells in comparison to ESCs (fold change > 1.5 and *t*-test *P* < 0.05).

The ESC-like cell lines showed the ability to differentiate spontaneously* in vitro* into derivatives of all three germ layers by EB formation at day 10 and after plating (Supplementary Figure 4 and Supplementary Figure 5A). mRNA and protein expression of the lineage specific marker genes for ectoderm (*Nestin*,* Map2*,* Tuj1*,* NeuN*,* GFAP*, and* Pax6*) (Supplementary Figure 4), mesoderm (*Gata4*,* Brachyury*,* EPCAM1*,* Myf5*,* MyoD*,* Islet1*,* SM-actin*, and* FLK1*), and endoderm (*Afp* and* Keratin-18*) demonstrate this (Supplementary Figure 5A). Moreover, directed differentiation into cardiomyocytes showed 14 (±3) beating areas for each well of 6-well plate with 87 (±36) beating contractions per minute (Supplementary Figure 3C; Supplementary film). Also differentiated cardiomyocytes were analyzed by whole-cell current clamp for pacemaker activity. It was observed that beating cells have a rhythmic action potential generation over time, with a constant amplitude (Supplementary Figure 3C).

Thus, the expression of lineage specific marker genes,* Map2*,* Nestin*,* NeuN*,* Pax6, Tuj1*, and* GFAP*, demonstrated the directed differentiation into the neural cell phenotype (Supplementary Figure 4). We further examined the function of ESC-like cell-derived neuronal cells by patch clamp recordings (Supplementary Figure 3F). Upon a brief current pulse of 5 ms, all cells (*n* = 6) could fire an action potential. There were cells that would fire continuously (2 cells out of 6 cells recorded) and cells that would not fire (4 cells out of 6 cells recorded, data not shown). Interestingly, the recorded cells displayed a strong hypopolarizing current after action potential generation. Moreover, the resting membrane potential was variable between the cells.

**Figure 9:**
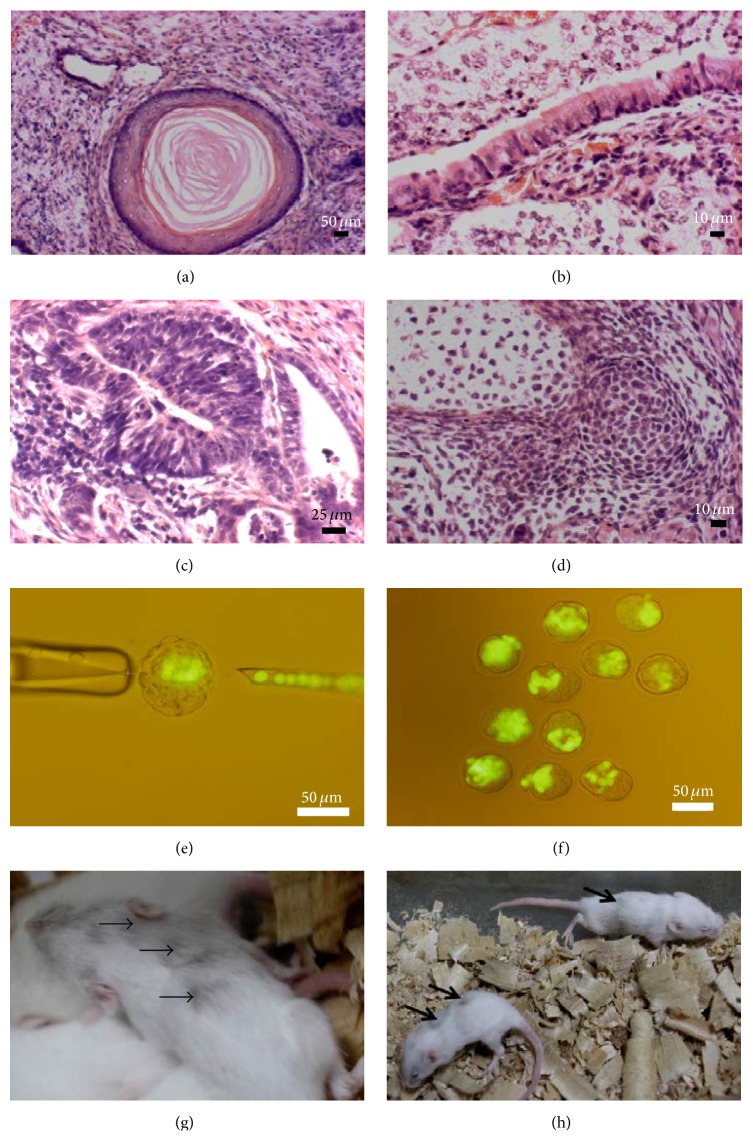
Pluripotency characterization of ESC-like cells with teratoma formation including tissue structures of all germ layers (a–d) and chimera formation (e–h). (a) Skin formation with keratinizing squamous epithelium, (b) respiratory epithelium with goblet cells, (c) neuronal rosette, and (d) primitive cartilage. (e, f) Blastocysts injected with GFP-marked ESC-like cells and chimera formation. Scale bars: (a), (e), and (f) 50 *μ*m, (b) and (d) 10 *μ*m, and (c) 25 *μ*m.

We tested the capacity of the ESC-like cells to form a teratoma and to generate chimeric mice to further confirm their pluripotency. We subcutaneously transplanted 2 × 10^6^ ESC-like cells into SCID mice. At four weeks after injection, ESC-like cells resulted in teratomas that contained all three germ layers (Figures 9(a)–9(d); Supplementary Figure 5B). Histological analyses showed the presence of neural structures and epidermis (as ectodermal derivatives), bone structure and adipose tissue (as mesodermal structures), and gut structure (as endodermal derivatives) in the teratoma sections (Figures 9(a)–9(d); Supplementary Figure 5B). To investigate chimera formation, ESC-like cells were transferred into blastocyst and chimeric mice were identified by coat color (Figures 9(e)–9(h)).

## 4. Discussion

SSCs are the only source of naturally occurring truly pluripotent stem cells in the organism after birth, which do not have to be artificially reprogrammed such as iPSCs. The molecular mechanisms underlying the natural shift from a unipotent to a completely pluripotent cell during the establishment of mouse ESC-like cells from SSCs are not completely understood until now. However, it appears that the age of animals, the mouse strain used, the culture conditions with growth factors involved, the cell density of SSCs during culture, the time period after initiation of culture, and the length of culture might all be key players in the transition process [5, 8–11].

In our work, we demonstrated the scarcity of the conversion of SSCs to ESC-like cells. Conversion occurred spontaneously from SSCs of neonate and up to 7-week-old mouse testis but not from older mice considered adult or even mature adult [12, 13]. This observation implies that the generation of ESC-like cells from SSCs coincides with the general development of the mouse up to an adolescent stage and thereafter ceases. Although mice are sexually mature by 35 days of age, relatively rapid maturational growth continues for most biological processes and cells. Tissues and organs continue to develop in the mouse until about three months of age [12, 13].

We observed that the amount of positive cells and signal density of Oct4-GFP in the seminiferous tubules of the neonate mouse testis was higher than in old mouse testis (after 7 weeks of age). Reduction of* Oct4, Nanog, Sox2*, and* TGFβ1* expression with aging may be related to a reduction of the undifferentiated SSC pool. These might include gonocytes and prespermatogonia in the testis of neonates and older animals. These observations might also indicate difficulties for the generation of ESC-like cells from older mice.

We also observed another limitation for appearance of ESC-like cells after initiation of culture that only occurred during a special time window (46 until 143 days) after initiation of SSCs cultures. Several reports concerning long-term cultivation for SSCs failed to describe this spontaneous shift of SSCs to pluripotent ESC-like cells [14, 15].

These results give the impression of a critical time window for the generation of pluripotent cells from SSCs and the impracticality of ESC-like cells being generated from continuous Oct4-GFP SSC culture. Kanatsu-Shinohara et al. also generated ESC-like cells during a time window about 4–7 weeks after initiation of culture in the neonate mouse SSCs [5]. To answer the question related to the origin of the SSC-ESC-like shift, different reporters including Stra8 [9], GPR125 [10], and Oct4 [7, 8] were employed for generation of ESC-like cells. Furthermore, Kanatsu-Shinohara et al. [16] analyzed the developmental fate of a single cell from a SSC culture that appeared during transfection experiments. But in all these experiments, the original cell source was heterogeneous, although the ESC-like cells were highly enriched.

Ko et al. [8] showed the induction of pluripotency from a SSC culture from Oct4 transgenic reporter mouse at postnatal day 35. They argued that this transition was mainly dependent on a distinct number of SSCs and on the length of culture for reprogramming (2–4 weeks), while they did not really mention if reprogramming occurred in every stage of the SSC culture.

We demonstrated that the ESC-like cells are fully pluripotent, express pluripotency markers, have the potential for complex teratoma formation, and produce chimera in the recipient mouse similar to mouse ESCs. Moreover, they are highly capable of differentiating into neuronal and cardiomyocyte phenotypes after* in vitro* differentiation, which also has been shown by other groups [17, 18].

It would be of major interest to study factors, including small molecules, that could increase the probability and also the restricted time window of SSC to PSC conversion. Recently it has been shown that the addition of glycogen synthase kinase-3 inhibitors to the testis-derived SC cultures increases the likelihood for the occurrence of ESC-like cells from SSCs [11].

In the primary culture of isolated cells from Oct4 transgenic reporter mice, we observed that the Oct4-GFP signal was expressed at a moderate level in neonate up to a low level in older or adult SSCs. This expression was completely downregulated during short- and long-term SSC culture [19], and a high density signal only remerged after conversion to ESC-like cells.

mRNA expression profiling confirmed that the expression of germ cell specific genes increased with age and was therefore significantly higher in SSCs from 7- to 12-week-old mice compared with neonatal SSCs. In parallel, we observed that the expression of* Oct4a* and* Nanog*,* and Sox2* was significantly upregulated in neonatal SSCs and downregulated in the adult SSCs.

In PSCs, core transcriptional genes control the expression of different lineage specific genes and prevent pluripotent cells from differentiation [20]. It has been demonstrated that these genes as well as other genes associated with pluripotency are already expressed in neonatal SCCs although mostly to a lower extent [5]. Our analysis showed a crucial time window for a shift of SSCs to ESC-like cells derived from mouse SSCs which occurred with a downregulation of germ cell genes and upregulation of core pluripotency genes in the postnatal mouse until adolescence. The natural potential of mouse SSCs to convert into a fully pluripotent cell comparable to mouse ESCs is still not understood. Some controversial challenges for pluripotency and multipotency of ESC-like cells exist (especially in ESC-like cells which were generated from human testis) [6]. However, the natural shift from a unipotent cell involved in spermatogenesis to a versatile cell population, which is able to differentiate into germ cells and cells of all germ layers, offers an ethically unproblematic and nonartificial alternative for regenerative medicine. But there might be limitations, which could be related to the age of the donor and also to a special time window in which natural reprogramming of SSCs can be observed during culture.

This study has come to the conclusion that the natural reprogramming of unipotent SSCs into pluripotent cells cannot occur during adulthood and implies that this conversion is only observable until adolescence and during a special time window after initiation of culture.

## Supplementary Material

In the supplementary method section, we describe in more detail the material and the methods of embryoid body (EB) formation, neuronal differentiation, cardiomyocyte differentiation, RNA extraction and RT-PCR analysis, gene expression analyses (Fluidigm Biomark), immunfluorescence staining (IMH), electrophysiology, FACS analysis, alkaline phosphatase assay, electron microscopy, production of teratoma and chimeric mice, and the statistical analysis.
