# Supplementary material for: Derivation of Pluripotent Cells from Mouse SSCs Seems to Be Age Dependent
Source: Stem Cells Int. 2015 Nov 9;2016:8216312. doi: 10.1155/2016/8216312 (PMC4655302; doi:10.1155/2016/8216312)

Supplementary Figures

Suppl. Figure 1: Different gene expression profiles of neonatal and adult (obtained from 7 and 12 weeks old mice) SSCs, ESC-like cells, mESCs and fibroblasts with germ cell-enriched and pluripotency associated genes.

(A) Dendogram, (B) Kohonen and (C) heatmap analysis clearly demonstrate that all subpopulation of cells localize in separated trees or areas.

Suppl. Figure 2: Characterization of ESC-like cells.

(A1-A3) Expression of high levels of Oct4-GFP, after conversion of SSC colonies to ESC-like cells.

(B) Flow cytometry analyses show that SSCs and ESC-like cells are highly positive for the cell surface markers CD9, CD29, CD49 and only weakly express CD117. In ESC-like cells GFRa1 is significantly down-regulated in comparison to SSCs. However, SSCs have a higher expression of CD9, CD29 and CD49 in comparison with testis cells. * $p<0.001$

Suppl. Figure 3: Pluripotency characterization of ESC-like cells.

(A) ESC-like cells, similar to mESCs, were strongly positive for alkaline phosphatase (AP).

(B1, B2) No expression of Oct4-GFP was observable in differentiated cardiomyocytes about 10 days after plating in. Beating cells are shown in Suppl. film.

(C) Electrophysiological analysis (patch clamp recording) in the beating area.

(D) In vitro differentiation of ESC-like cells into neuronal cells with expression of glial and neuronal markers.

(F) Action potential for Na+ and K+ channel in differentiated neuronal cells (E).

Suppl. Figure 4: Pluripotency characterization of ESC-like cells. In vitro differentiation of ESC-like cells into neuronal cells with the expression of different neuronal markers and GFAP.

Suppl. Figure 5: Pluripotency characterization of ESC-like cells. (A) RT-PCR analysis for expression of mesodermal, ectodermal and endodermal lineages genes in mESCs, CF-1 MEF, cells, Early differentiation (6-10 days after plating), EB formation for cardiomyocte cells (EB-CD) at day 10, EB formation for neuronal cells (EB-ND) at day 10 and water control. Overview of complex teratoma derived from ESC-like cells (B).

Suppl. Film: Beating area after cardiomyocyte differentiation of ESC-like cells.

Suppl. Figure 1:

A

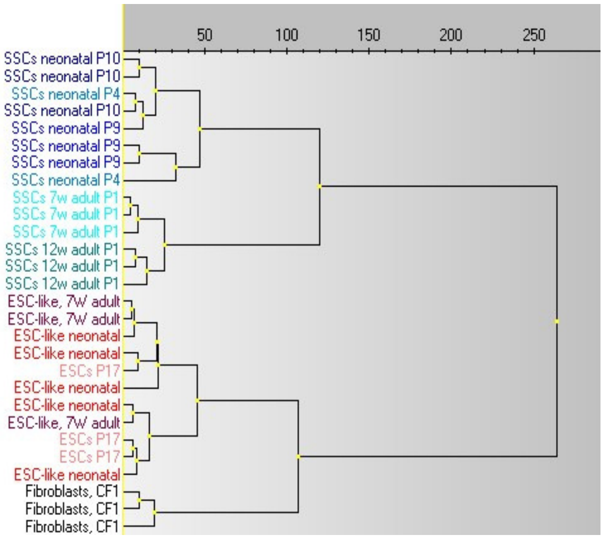

B

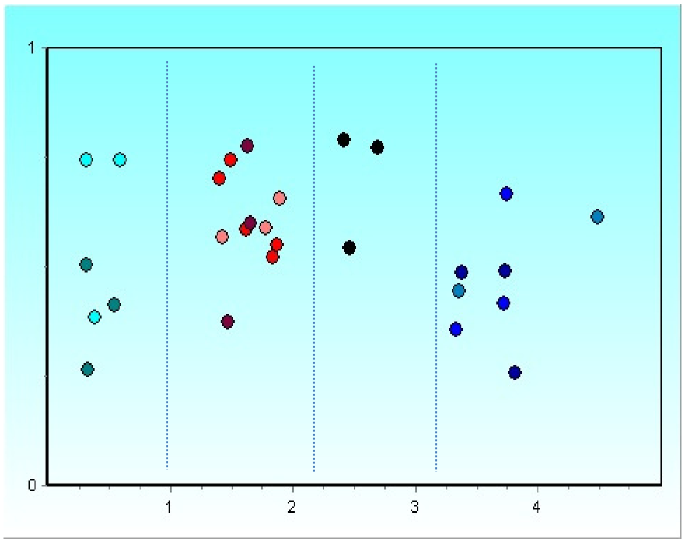

C

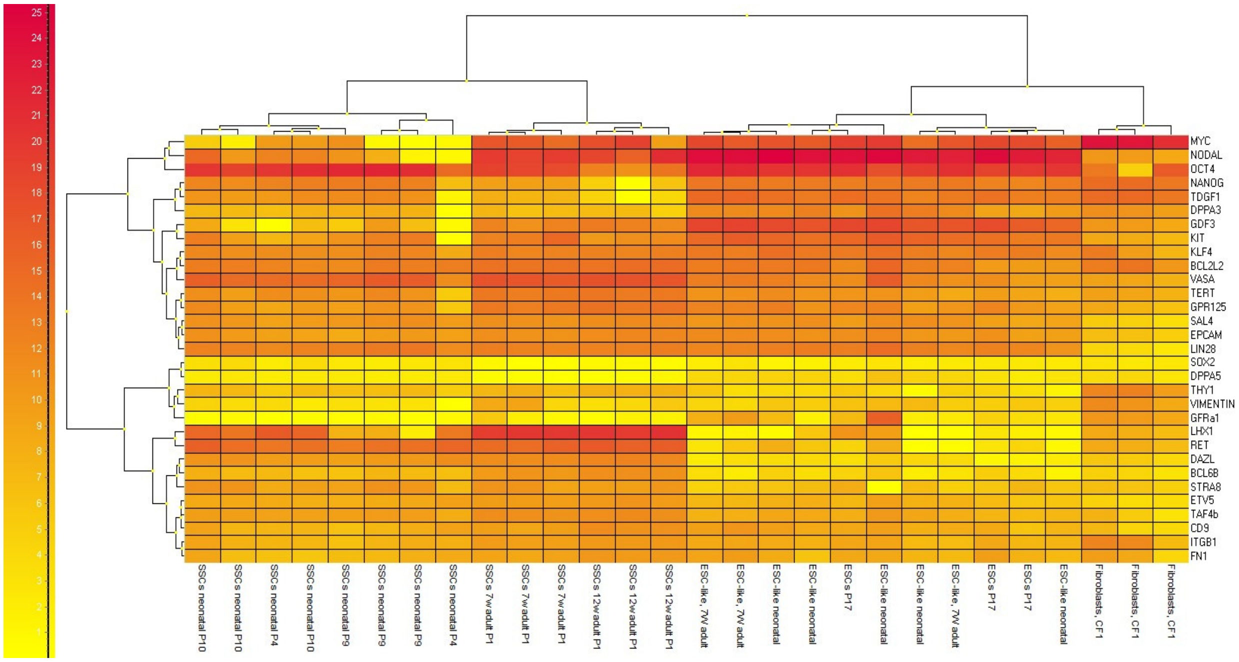

Suppl. Figure 2:

Suppl. Figure 2:

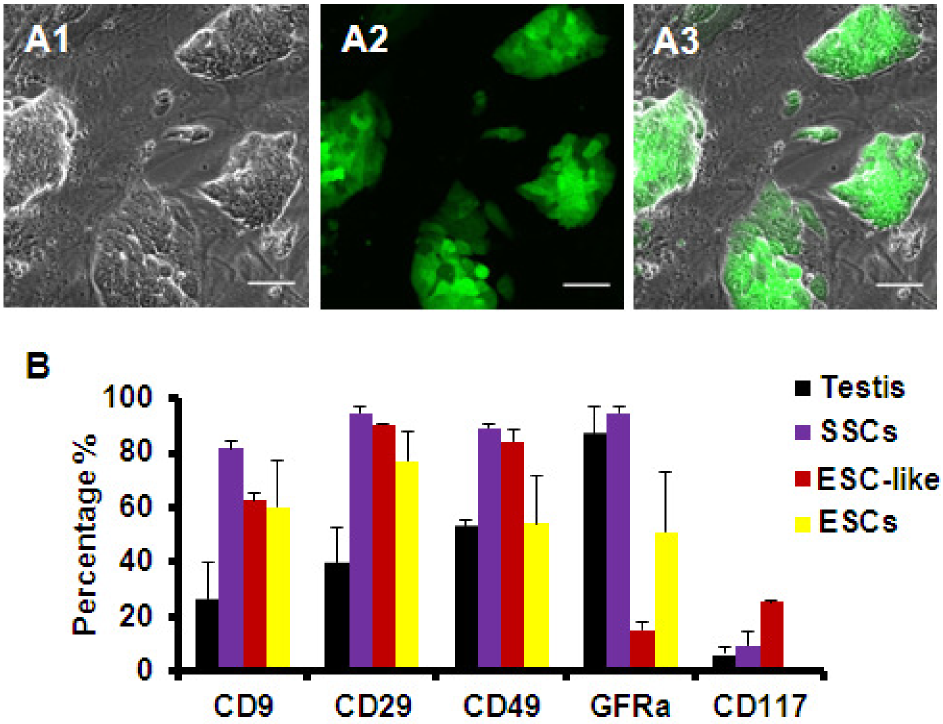

Suppl. Figure 2:

Suppl. Figure 2:

Suppl. Figure 3:

:

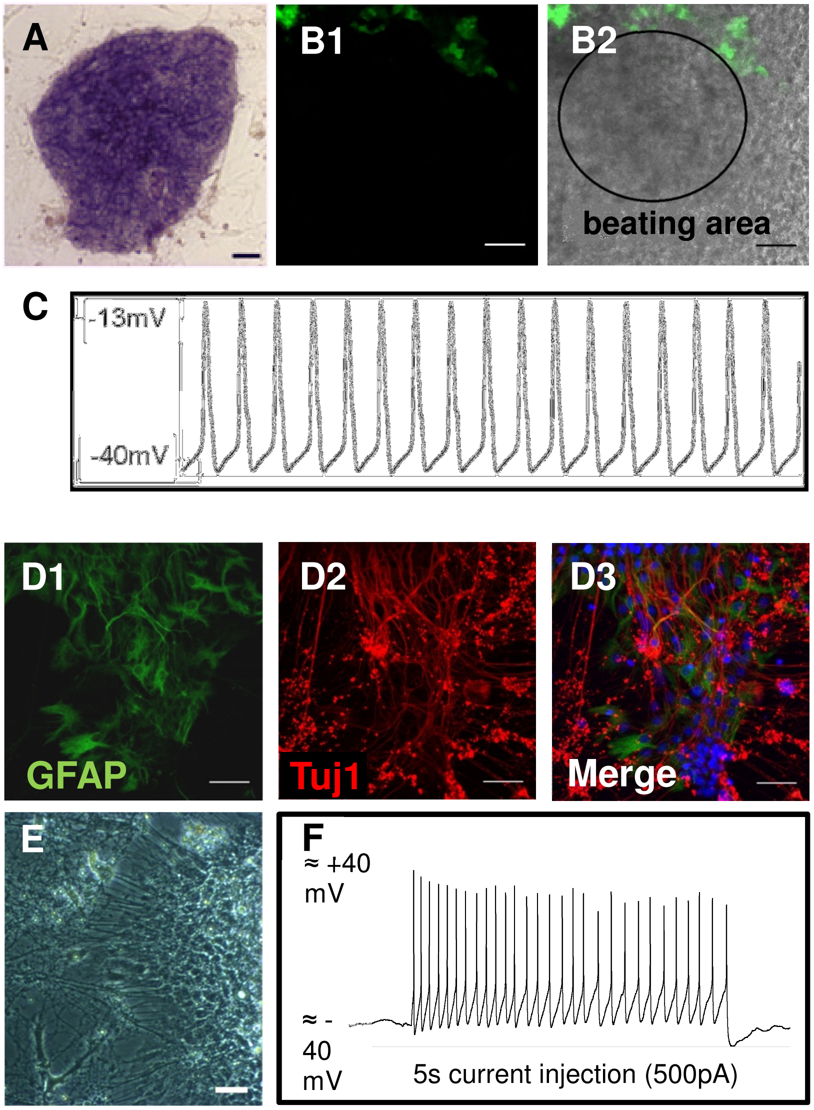

Suppl. Figure 4:

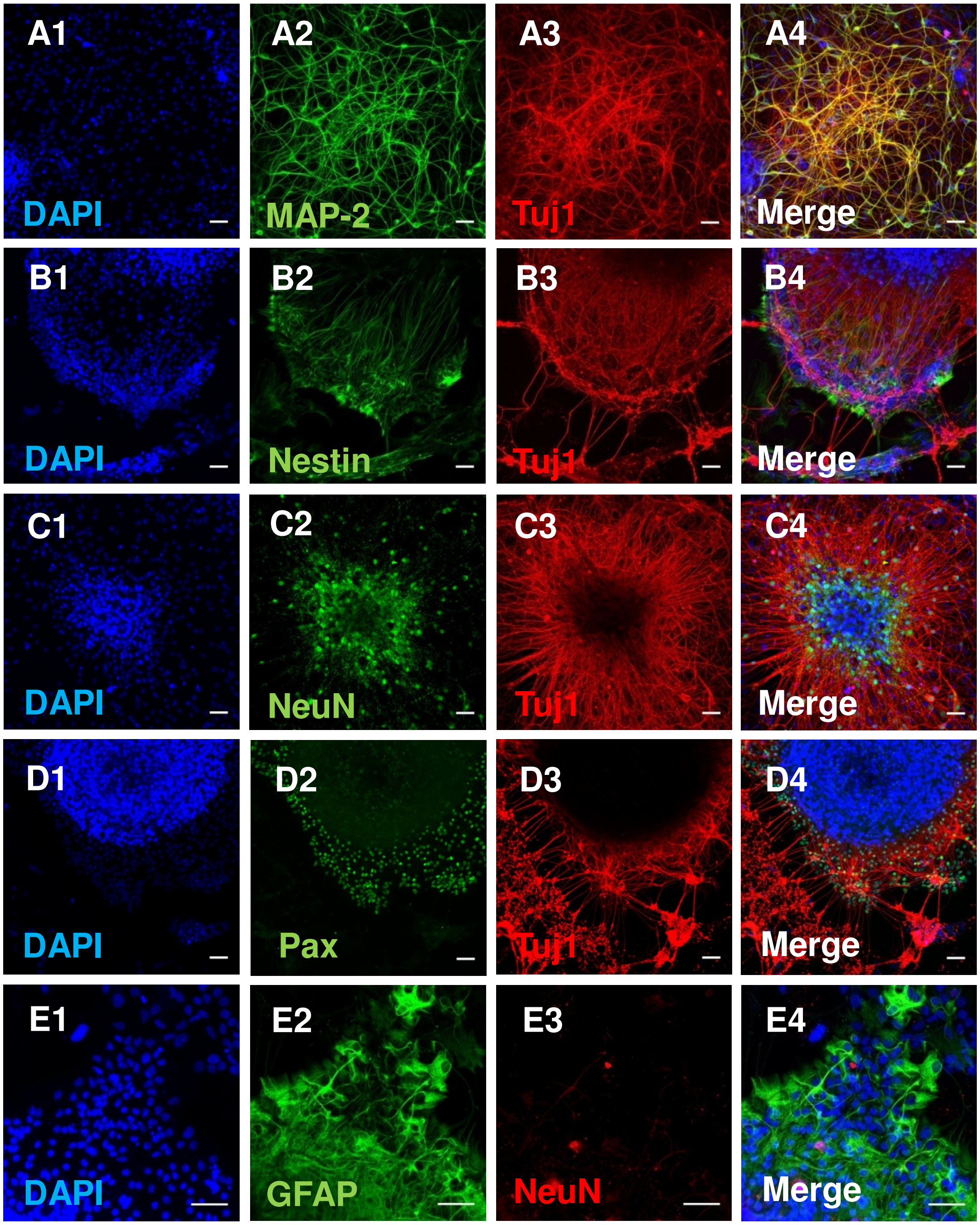

Suppl. Figure 5:

A

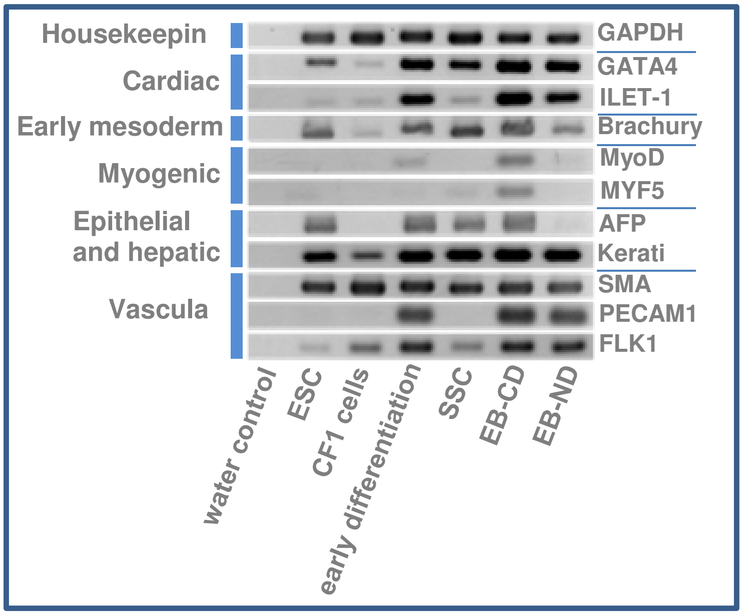

B

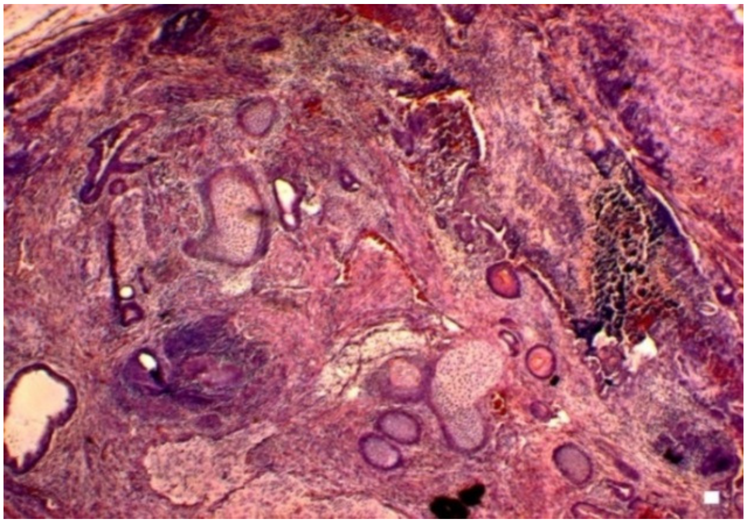

Supplement: Supplementary file 1 — In the supplementary method section, we describe in more detail the material and the methods of embryoid body (EB) formation, neuronal differentiation, cardiomyocyte differentiation, RNA extraction and RT-PCR analysis, gene expression analyses (Fluidigm Biomark), immunfluorescence staining (IMH), electrophysiology, FACS analysis, alkaline phosphatase assay, electron microscopy, production of teratoma and chimeric mice, and the statistical analysis. [file 8216312.f1.zip › mat.646972.v2/646972.supp/mat.646972.v1.pdf]
