# Supplementary material for: Derivation of Pluripotent Cells from Mouse SSCs Seems to Be Age Dependent
Source: Stem Cells Int. 2015 Nov 9;2016:8216312. doi: 10.1155/2016/8216312 (PMC4655302; doi:10.1155/2016/8216312)
Supplement: Supplementary file 1 — In the supplementary method section, we describe in more detail the material and the methods of embryoid body (EB) formation, neuronal differentiation, cardiomyocyte differentiation, RNA extraction and RT-PCR analysis, gene expression analyses (Fluidigm Biomark), immunfluorescence staining (IMH), electrophysiology, FACS analysis, alkaline phosphatase assay, electron microscopy, production of teratoma and chimeric mice, and the statistical analysis. [file 8216312.f1.zip › mat.646972.v2/646972.supp/Supplementary Tables.docx]

**Supplementary Tables**

**Tables 1:**

***t-test of SSCs from neonatal mice vers. SSCs from 7-12 week old adult mice***

| (SSCs neonatal) vs (SSCs adult 7-12w) | Fold change |
| --- | --- |
| BCL6B | -15,07508 |
| DPPA5 | 3,1818 |
| SOX2 | 4,74169 |
| TAF4b | -3,57527 |
| ETV5 | -2,94365 |
| ITGB1 | -4,95634 |
| BCL2L2 | -3,11793 |
| DAZL | -2,91417 |
| GPR125 | -16,35907 |
| MYC | -1830,23506 |
| GDF3 | -140,13906 |
| FN1 | -5,49775 |
| EPCAM | -3,3626 |
| NODAL | -1097,19921 |
| CD9 | -5,27345 |
| LHX1 | -354,59006 |
| TERT | -9,07858 |
| NANOG | 36,44733 |
| VASA | -4,39831 |
| OCT4 | 22,29543 |
| SAL4 | -1,82736 |
| VIMENTIN | -7,43638 |
| RET | -2,405 |
| KLF4 | -2,46455 |
| THY1 | -2,16232 |
| TDGF1 | 21,80762 |
| GFRa1 | -2,65057 |
| KIT | -6,87127 |
| STRA8 | -1,30725 |
| LIN28 | -1,03047 |
| DPPA3 | 1,11228 |

***t-test of SSCs from 7 week old adult mice vers. SSCs from 12 week old adult mice***

| (SSCs neonatal) vs (SSCs 12w adult P1) | Fold change | Difference (A-B log scale) | P-Value |
| --- | --- | --- | --- |
| BCL6B | -19,86367 | -4,31206 | 0,000001018 |
| DPPA5 | 2,91652 | 1,54425 | 0,000007193 |
| ETV5 | -3,53909 | -1,82338 | 0,00011195 |
| TAF4b | -3,84286 | -1,94218 | 0,000324889 |
| OCT4 | 172,44036 | 7,42995 | 0,000541481 |
| SOX2 | 4,17131 | 2,0605 | 0,000549374 |
| CD9 | -9,42156 | -3,23597 | 0,000552608 |
| ITGB1 | -5,94606 | -2,57193 | 0,000657148 |
| DAZL | -3,75497 | -1,9088 | 0,000778097 |
| NANOG | 272,77374 | 8,09156 | 0,000789102 |
| FN1 | -8,53648 | -3,09364 | 0,001232242 |
| BCL2L2 | -3,67151 | -1,87637 | 0,001516725 |
| EPCAM | -4,47245 | -2,16107 | 0,001614133 |
| GPR125 | -21,02807 | -4,39424 | 0,003583854 |
| GDF3 | -166,15548 | -7,37639 | 0,007690741 |
| RET | -3,59744 | -1,84697 | 0,009077612 |
| MYC | -1436,40401 | -10,48825 | 0,010937024 |
| TDGF1 | 117,54811 | 6,87711 | 0,013131952 |
| NODAL | -824,91913 | -9,68811 | 0,015598175 |
| SALL4 | -2,19932 | -1,13706 | 0,01851919 |
| LHX1 | -391,58371 | -8,61318 | 0,019909393 |
| THY1 | -2,63146 | -1,39587 | 0,027894775 |
| GFRa1 | -1,50513 | -0,58989 | 0,030103544 |
| TERT | -9,82556 | -3,29654 | 0,030892417 |
| VASA | -4,89286 | -2,29068 | 0,039340369 |
| KLF4 | -2,48523 | -1,31338 | 0,098597143 |
| VIMENTIN | -2,6432 | -1,40229 | 0,136152634 |
| KIT | -2,89921 | -1,53566 | 0,566802525 |
| DPPA3 | 1,41449 | 0,50028 | 0,771087752 |
| STRA8 | -1,15131 | -0,20327 | 0,8190814 |
| LIN28 | -1,06068 | -0,08499 | 0,847259705 |

***t-test analysis of ESC-like cells vers. mESCs***

| (ESC-like) vs (ESCs P17) | Fold change | Difference (A-B log scale) | P-Value |
| --- | --- | --- | --- |
| NANOG | 1,72811 | 0,7892 | 0,001341143 |
| DPPA3 | 5,70353 | 2,51186 | 0,014558404 |
| SALL4 | 2,60971 | 1,38389 | 0,017143882 |
| BCL2L2 | 5,40657 | 2,43471 | 0,024183204 |
| DPPA5 | 2,97558 | 1,57317 | 0,025458076 |
| DAZL | 3,06788 | 1,61724 | 0,052857757 |
| ETV5 | 2,09402 | 1,06627 | 0,124009187 |
| MYC | -3,00936 | -1,58945 | 0,124856236 |
| STRA8 | -4,3106 | -2,10789 | 0,150482399 |
| TAF4b | 1,87021 | 0,9032 | 0,154860984 |
| CD9 | 2,20338 | 1,13972 | 0,193950046 |
| KIT | 1,9764 | 0,98288 | 0,194262911 |
| VASA | 3,03848 | 1,60335 | 0,220106473 |
| BCL6B | -2,25121 | -1,1707 | 0,254277601 |
| LHX1 | -6,44816 | -2,68889 | 0,320601152 |
| FN1 | -1,68699 | -0,75446 | 0,402469231 |
| THY1 | -1,92702 | -0,94637 | 0,404828882 |
| SOX2 | -1,17939 | -0,23805 | 0,502098613 |
| EPCAM | 1,29439 | 0,37228 | 0,549262057 |
| ITGB1 | -1,27075 | -0,34568 | 0,580390453 |
| TERT | 1,31309 | 0,39297 | 0,632159326 |
| OCT4 | 1,29866 | 0,37702 | 0,651045397 |
| GFRa1 | 2,13959 | 1,09733 | 0,728602204 |
| GPR125 | -1,25349 | -0,32595 | 0,735276329 |
| VIMENTIN | -1,1431 | -0,19295 | 0,792662299 |
| TDGF1 | -1,03802 | -0,05384 | 0,936875619 |
| RET | 1,09254 | 0,12769 | 0,951083916 |
| KLF4 | -1,00536 | -0,00771 | 0,989931778 |
| GDF3 | 1,00592 | 0,00851 | > 0.99 |
| LIN28 | -1,00184 | -0,00265 | > 0.99 |
| NODAL | 1,00018 | 0,00026 | > 0.99 |

**Tables 2:**

***Correlation analysis of neonatal SSCs vers. SSCs from 12 week old adult mice***

***Correlation analysis of SSCs from 7 week old adult mice vers. SSCs from 12 week old adult mice***

***Correlation analysis of ESC-like cells vers. mESCs***
